# Stability and Longevity in the Publication Careers of U.S. Doctorate Recipients

**DOI:** 10.1371/journal.pone.0154741

**Published:** 2016-04-29

**Authors:** Cathelijn J. F. Waaijer, Benoît Macaluso, Cassidy R. Sugimoto, Vincent Larivière

**Affiliations:** 1 Centre for Science and Technology Studies, Faculty of Social and Behavioural Sciences, Leiden University, Leiden, the Netherlands; 2 Observatoire des sciences et des technologies, Centre interuniversitaire de recherche sur la science et la technologie, Université du Québec à Montréal, Montréal, Canada; 3 School of Informatics and Computing, Indiana University, Bloomington, Indiana, United States of America; 4 École de bibliothéconomie et des sciences de l’information, Université de Montréal, Montréal, Canada; Universidad de Las Palmas de Gran Canaria, SPAIN

## Abstract

Since the 1950s, the number of doctorate recipients has risen dramatically in the United States. In this paper, we investigate whether the longevity of doctorate recipients’ publication careers has changed. This is achieved by matching 1951–2010 doctorate recipients with rare names in astrophysics, chemistry, economics, genetics and psychology in the dissertation database ProQuest to their publications in the publication database Web of Science. Our study shows that pre-PhD publication careers have changed: the median year of first publication has shifted from after the PhD to several years before PhD in most of the studied fields. In contrast, post-PhD publication career spans have not changed much in most fields. The share of doctorate recipients who have published for more than twenty years has remained stable over time; the shares of doctorate recipients publishing for shorter periods also remained almost unchanged. Thus, though there have been changes in pre-PhD publication careers, post-PhD career spans remained quite stable.

## Introduction

The career system in modern day academia is typically pyramidal in structure with relatively few professors at the top and many PhD students at the bottom. The shape of this pyramid may differ by country, the United States for example having relatively more researchers in its highest academic position than Germany [1]. In most cases, supply exceeds demand: more PhD students and postdoctoral researchers indicate they would like to have an academic research career than there are positions available [2–4]. Indeed, studies have shown that opportunities to occupy tenured or tenure track faculty positions in academia have decreased, as the number of such positions has not kept track with the number of doctorates awarded [4–8].

The concomitant growth in doctoral students and decline in the growth rate of tenure track positions have raised concerns about the ethics of doctoral education. For example, Ioannidis, Boyack, and Klavans [9], found that only 1% of the scientific workforce is continually publishing and admonished the educational system for utilizing doctoral students as “a cheap workforce for materializing resource-intensive incremental research agenda.” They assert that this research system “may be exploiting the work of millions of young scientists for a number of years without being able to offer continuous, long-term stable investigative careers to the majority of them.” This assertion, however, is relatively untested. Although the proportion of doctoral graduates obtaining tenure track jobs in academia is decreasing [10], “investigative careers” can be found in a number of positions within the scientific workforce. Systematic investigation, therefore, is necessary to ascertain the proportion of doctoral students who continue to contribute to the advancement of knowledge, regardless of position status and type.

Many doctoral students contribute to scholarship during their doctoral training. For example, in a study of Québec scholarship, it was noted that students were authors on a third of academic articles [11]. Publication during the doctoral program was correlated with degree completion and continuing productivity. There are, however, no equivalent studies of the degree to which students in the United States participate in scholarship and when, relative to the conferral of the doctoral degree, they first publish. Furthermore, we lack information on the length of the publishing careers for these individuals. Concerns about the stability of the investigative careers are often linked with the conversations around the erosion of tenure in the United States. That is, have the changes in the composition of the academic workforce replaced long investigative careers with more volatile short-term and temporary positions?

The present study provides a novel perspective by empirically measuring the investigative careers of U.S. doctoral students. Rather than analyzing the proportion of doctoral graduates entering into various career types—as previous studies have performed [4, 12]—this study focuses on the proportion of doctoral graduates who publish and the length of their publication careers. To analyze variation by both time and field, we analyze doctoral graduate from 1951–2010 across five fields (astrophysics, chemistry, economics, genetics, and psychology). We seek to address the following three research questions:

1. How has the proportion of doctoral graduates who publish academic articles changed from 1951 to now?
2. How has the year in which doctoral students publish their first academic article changed through time?
3. How has the length of the investigative career for doctoral graduates changed through time?

The strength of the scientific workforce directly translates to improved economic, health, and social well-being. For a country to be scientifically competitive, it needs to maximize its human intellectual capital-base and invest in this resource. As stated in the Science and Engineering Indicators [13], “the pursuit of new knowledge, the training of the people in whom it is embodied, and its exploitation toward generating innovation, makes academia a national resource whose vitality rests in the scientists and engineers who work there”. In order to better understand this national resource, it is imperative that we replace anecdote with evidence—specifically, with a more nuanced understanding of the contribution of doctoral graduates to the advancement of knowledge and the stability of publishing careers for these individuals.

## Data and methods

### Data

#### ProQuest database

The ProQuest Dissertations and Theses database was used as a source of data on U.S. doctorate recipients. This database, provided by ProQuest LLC, offers “comprehensive historic and ongoing coverage for North American works” [14]. Evidence of the comprehensiveness of the database is provided by the fact that the U.S. Library of Congress, which “strives to hold copies of all U.S. doctoral dissertations”, uses the ProQuest database as an indexing service of U.S. doctoral dissertations [15].

The data were provided in XML format in March 2012 and cover over 2.3 million dissertations completed between 1743 and 2012. Most of these are doctoral dissertations (rather than other types of theses) [16] and a large majority have been written at U.S. institutions [17]. A total of 1,668,925 dissertations completed in the course of a research doctorate from a U.S. institution are indexed in our version of the ProQuest database [16]. To determine the suitability of the ProQuest database as a source of U.S. doctorate recipients, we compared the number of research doctorates indexed in ProQuest by year to the number of research doctorates according to NSF data [18, 19]. This comparison shows that numbers are very similar, especially from the mid-1960s (S1 Fig, panel A). A true measure of coverage would compare whether doctorate recipients in one source are also in the other source, and vice versa, but here such an analysis is not possible. Thus we have to limit ourselves to measuring the ratio between the number of research doctorates indexed in ProQuest and the number given by the NSF. This ratio shows that the number of research doctorates indexed by ProQuest is only 20% for the start of the 1950s, but quickly increases to over 90% for the years after 1965 (S1 Fig, panel B). Although “coverage” of the ProQuest database may be lower in the 1950s, trends in the increase and decrease of doctorate recipients are mirrored closely.

The research field(s) in which the doctorate has been completed is also stored in ProQuest. ProQuest distinguishes 165 “disciplines” (major fields), such as anthropology, biology, physics, and sociology. These disciplines are divided into 432 “specialties” (subfields). Five disciplines/specialties were selected for our analysis: astronomy and astrophysics (specialty; in the remainder of the paper shortened to “astrophysics”), chemistry (discipline), economics (discipline), genetics (specialty), and psychology (discipline). Thus the selection includes fields from the natural, life, and social sciences. We chose fields with a considerable number of doctorate recipients from the 1950s, so as to make longitudinal analysis possible. This criterion meant that a field like molecular biology, where the number of PhD graduates was in the single digits until the late 1970s, could not be selected. The selected fields also satisfy the criterion of good coverage by the WoS [20]. Astrophysics, economics, and genetics are all basic research fields, in which a PhD degree is mostly the gateway towards a research career [21]. On the other hand, industry is a major post-PhD sector of employment for chemistry and psychology doctorate recipients; in the former are employed in the chemical industry and in the latter, many doctorate recipients are involved in clinical work [21].

Furthermore, all selected fields are relatively monodisciplinary and have a set of core journals. To prevent false positives we introduced the condition that at least one of the papers published by a PhD graduate should be in the field of their dissertation (see “Retrieval of papers” section). For example, someone may have received a PhD from a neurology department and thus be listed as a neurology PhD graduate, but have only published papers in molecular biology journals.

The number of doctorate recipients varies heavily by field: the largest field is psychology, followed by chemistry and economics according to ProQuest (S2 Fig). Genetics and astrophysics, as specialties instead fields according to ProQuest, were smaller. As expected [22], the conferral of doctoral degrees in all fields showed large increases after the 1950s.

#### Web of Science database

The Web of Science (WoS), a large bibliographic database that covers a period from 1900 to the present, was used as a source of scientific articles. The WoS version we used is maintained at the Observatoire des Sciences et des Technologies (OST), Université de Québec à Montréal (UQAM), Canada, and contains the Science Citation Index Expanded (SCIE), the Social Science Citation Index (SSCI) and the Arts and Humanities Citation Index (AHCI). This database employs NSF’s Science and Technology Indicators journal classification of two levels for research fields [23], which divides publications into twelve disciplines. These disciplines are further subdivided into 134 specialties. As Moed describes in his handbook on citation analysis, the WoS covered approximately 7,500 scientific journals in 2005 [20], which were selected to cover the most important scholarly communication. However, coverage varies by discipline–as the use of journals for scholarly communication is different in various disciplines–and so does the inclusion of journals in the WoS.

In this study, the determination of publication career spans and the first year of publication is limited to the WoS. It should be kept in mind that in earlier years, fewer journals were indexed in the database (S3 Fig), which may lead to a smaller likelihood of publications being included in our analysis than in recent times. At the same time, fewer scientists were active in the earlier period [24]. Assuming the increase in the number of journals follows the increase in the number of scientists, the expansion of the WoS does not affect our results. Indeed, the growth in the number of journals in the database (S3 Fig) has been quite similar to the growth in the number of U.S. doctorate recipients (S2 Fig). In chemistry and psychology, the large growth in both the number of doctorate recipients and the number of journals in the 1990s is especially apparent in the figure. On the other hand, the growth in the number of journals in, particularly, economics and psychology from 2007 is not matched by an increase in the number of doctorate recipients–a fact that should be kept in mind especially when interpreting the share of doctorate recipients publishing one or more papers (see Results section).

### Linking ProQuest and WoS

#### Linking strategy

In order to measure the year of first publication and the publication span of doctorate recipients, publications should be attributed correctly to each recipient. However, in practice, a publication may not be attributed to a person having authored that publication (false negative) or a publication may be attributed to a person who has not authored that publication (false positive). False negatives can result from spelling variants or errors or name changes (e.g., due to marriage; [25]). False positives mainly result from homonymic names: names shared by multiple persons.

In our study, we are mainly concerned with false positives due to homonyms, as they erroneously lengthen a doctorate recipient’s publication career. False negatives due to spelling variants and errors, are less problematic, because we are interested in the *first* publication and the publication career *spans* of these doctorate recipients. Missing publications only affect the year of first publication if a person’s first publication happens to be missed and a doctorate recipient published only one paper in that year. They only affect publication career spans if a person’s last publication is missed. As such false negatives due to spelling variants and errors occur randomly, they do not bias our results. Only in the case that doctorate recipients start publishing under a different name, will missing publications result in a shorter publication career than they have actually had. This may occur for women that get married or divorced after their first publication and publish under a different name after this marriage or divorce. Unfortunately, we cannot correct for this in our data, and it is difficult to estimate its precise effect. Several factors could have been at play and have had opposing effects. First of all, the share of women has increased over time [26]. We measured how much this share increased in our dataset using gender assignment of the first names of doctorate recipients (as described in [27]; S1 Table). This is likely to have led to more false negatives over time. In addition, women marry later in life, which also leads to more false negatives over time [28]. However, this only occurs if they also change their name on publications when they get married, which we expect them to do less in recent times than fifty to sixty years ago [29]. This leads, in turn, to fewer false negatives. Finally, marriage rates have fluctuated over the course of the study period [30], so the number of women doctoral students and doctorate recipients to have gotten married *at all* will have fluctuated, too. This makes it difficult, if not impossible, to estimate the overall effect of longitudinal trends in gender and name changes on false negatives in our study.

There are several options for constructing a dataset of doctorate recipients and the papers they have authored: manual homonym disambiguation of the papers authored by the entire group of doctorate recipients, manual disambiguation of a sample, automatic disambiguation, or restricting the sample to authors with unique names. The first, manual disambiguation of the entire group, was impossible due to the large number of doctorate recipients in our study. The second would have been practically feasible but still very time-consuming. Furthermore, manual disambiguation is likely to be easier for more recent doctorate recipients, as they may be more traceable online. It is therefore likely that incorrect attribution would be more prevalent for the early group of doctorate recipients, which could introduce a bias in longitudinal analyses. The third option of automatic disambiguation heavily relies on “seed” publications (publications that one is sure are authored by the researcher, e.g., [31, 32]) or on email addresses for disambiguating very common names (e.g., [33]). For this study, we do not know which publications are unambiguously authored by a doctorate recipient (in fact, we do not know if they even *have* authored a WoS-indexed publication) and publications authored before 2004 do not have email addresses, so automatic disambiguation would be difficult, if not impossible.

Therefore, we decided to use a pragmatic approach by restricting our analysis to authors with unique names. Such an approach has also been followed by Boyack and Klavans [34], *inter alios*. This approach has the advantage that it makes matching of author names from ProQuest to papers in the WoS possible while still obtaining unbiased results, as it is unlikely that a person with a unique name would behave any differently from a person with a common name. Below, we describe how unique names were selected.

#### Selection of unique names

Variation in surnames and combinations of surnames and initials varies by country. For example, the ten most frequent surnames in the U.S. and Norway account for less than 5% of the population, whereas in Korea the distribution is more skewed [35, 36]. When including initials, names become much more unique. In Norway, 86% of publishing researchers have a unique combination of surname plus initial(s), whereas only about a third of researchers in the Canadian province of Québec have a unique combination [35, 37].

In order to reduce the likelihood of homonyms, we selected names of doctorate recipients that occurred only once in ProQuest. This means that doctorate recipients from linguistic and cultural groups that have a larger share of common names (e.g., from Eastern Asia; [35]) are likely to be underrepresented in our sample. A further selection criterion to reduce the likelihood of homonyms was to only select names with two or three initials [38]. As a next step, surnames occurring commonly in the Web of Science were removed (i.e., for each surname the number of distinct combinations of surname and initials was counted, and surnames occurring in 100 or more combinations were removed). Finally, we removed names of researchers with publications in fields distinct from that of the PhDs, as such publications suggests these names are homonyms. However, it would be too restrictive to remove all names with publications outside the precise field of PhD: many researchers publish in adjacent fields. Therefore, only the names with publications outside the broader research field were removed. In S2 Table we show how we defined the broader research field for each group of doctorate recipients.

#### Retrieval of papers

Unique names in the ProQuest database need not be unique in the much larger WoS database. Therefore, further criteria were imposed on the retrieval of papers from this database. They concern several dimensions: the type of papers, the period for which papers were retrieved, the year of first publication, and the field of publication.

The type of paper was limited to “articles” or “reviews” as we are interested in measuring the research output of doctorate recipients. Papers published between five years before PhD and thirty years thereafter were retrieved. This period was chosen because publications published long before the PhD (e.g., ten years before PhD), or many years after (e.g., sixty years after) are unlikely to be authored by the doctorate recipient. In some cases, a thirty year limit might be too restrictive; however, in our main outcome measure, the span of the research career after the PhD, all publishing for over twenty years is considered to be a “life-long” career. As a further selection criterion a doctorate recipient’s first publication must be between five years before and three or five years after PhD graduation; three years for astrophysics, chemistry, and genetics; five years for economics and psychology as PhD graduates in these fields publish their first paper later than in the other three fields (see the results section on year of first publication). In addition, at least one publication must be in the (narrow) field of their PhD. The tables of selected doctorate recipients and their publications used for the analyses of publication careers were constructed using the version of the WoS database of 2 October 2015.

#### Robustness of unique name selection

The robustness of the selection of unique names was tested by determining the effect of each selection step on the number of excluded doctorate recipients (S4 Fig; panel A) and on the *mean* number of papers published by the doctorate recipients (S4 Fig; panel B; [39]). This analysis showed that the main effect on both the number of doctorate recipients and the mean number of publications came from the very first selection step: selection from the entire set of U.S. doctorate recipients to only those with a name that is unique in ProQuest. Subsequent steps of taking only doctorate recipients with two or three initials, with a surname that is not very frequent in the WoS, with a name that does not have publications in a disparate domain, and a first publication too long after the year of the doctorate did reduce the number of included doctorate recipients and their mean number of publications, but not by as much as the first selection step. The fact that the first selection step heavily reduced the mean number of publications indicates that this was the main step by which homonymic names were removed. With the subsequent selection steps, we further refined our selection. After the final selection step, the sample consisted of 30% of U.S. doctorate recipients in astrophysics, 30% in chemistry, 26% in economics, 29% in genetics, and 30% in psychology. These shares were quite stable in all fields, except for economics, where the share went down from 30% in the 1960s to 19% in 2006–2010 (S3 Table).

#### Statistical analysis

Statistical analyses were performed in R i386 3.2.3 using the packages stats for Kruskal Wallis rank sum tests and glm for logistic regression. The package ggplot2 was used to make figures.

## Results

### Share of doctorate recipients with one or more publications

**Fig 1:**
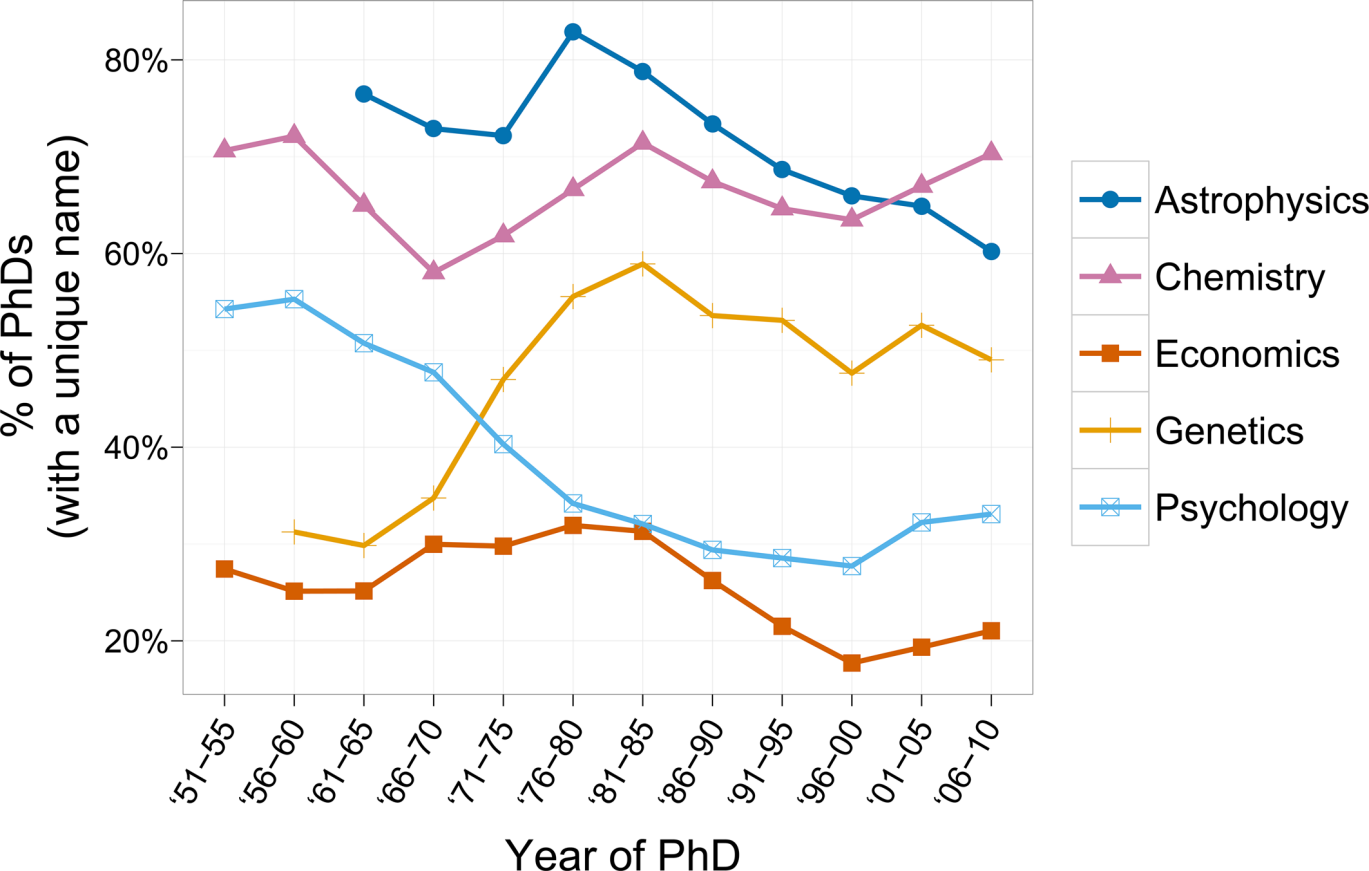
Percentage of doctorate recipients with at least one publication. Datapoint only shown when number of doctorate recipients with a unique name in a given period > 100.

First, we investigated the share of doctorate recipients with one or more publications. Fig 1 shows the shares of doctorate recipients who have published at least one paper, by year of PhD (in five-year time periods). There are large variations by discipline, with economics at the low end (25% over all time) and astrophysics at the high end (69% over all time; Fig 1). This suggests that the entrance into the scholarly communication system by doctorate recipients in the U.S. varies heavily by field, with publishing being the norm in astrophysics, chemistry and genetics, but not in economics [11]. The proportion of doctorate recipients associated with publications has not, however, remained stable across time. As shown, rates have declined in astrophysics, economics, and psychology, have risen in genetics, and have fluctuated over time in chemistry.

A possible explanation for the fact that in three of the five fields the rates declined could be that admission into doctoral programmes may have become less selective as the number of doctoral enrollments grew. The increase in chemistry, economics and psychology from 2001 may have been of a more methodological nature: as shown in the Data and methods section, the number of journals indexed in the WoS grew in these disciplines, thereby increasing the chance of doctorate recipients in these fields to publish a paper indexed in the WoS.

### Year of first publication

**Fig 2:**
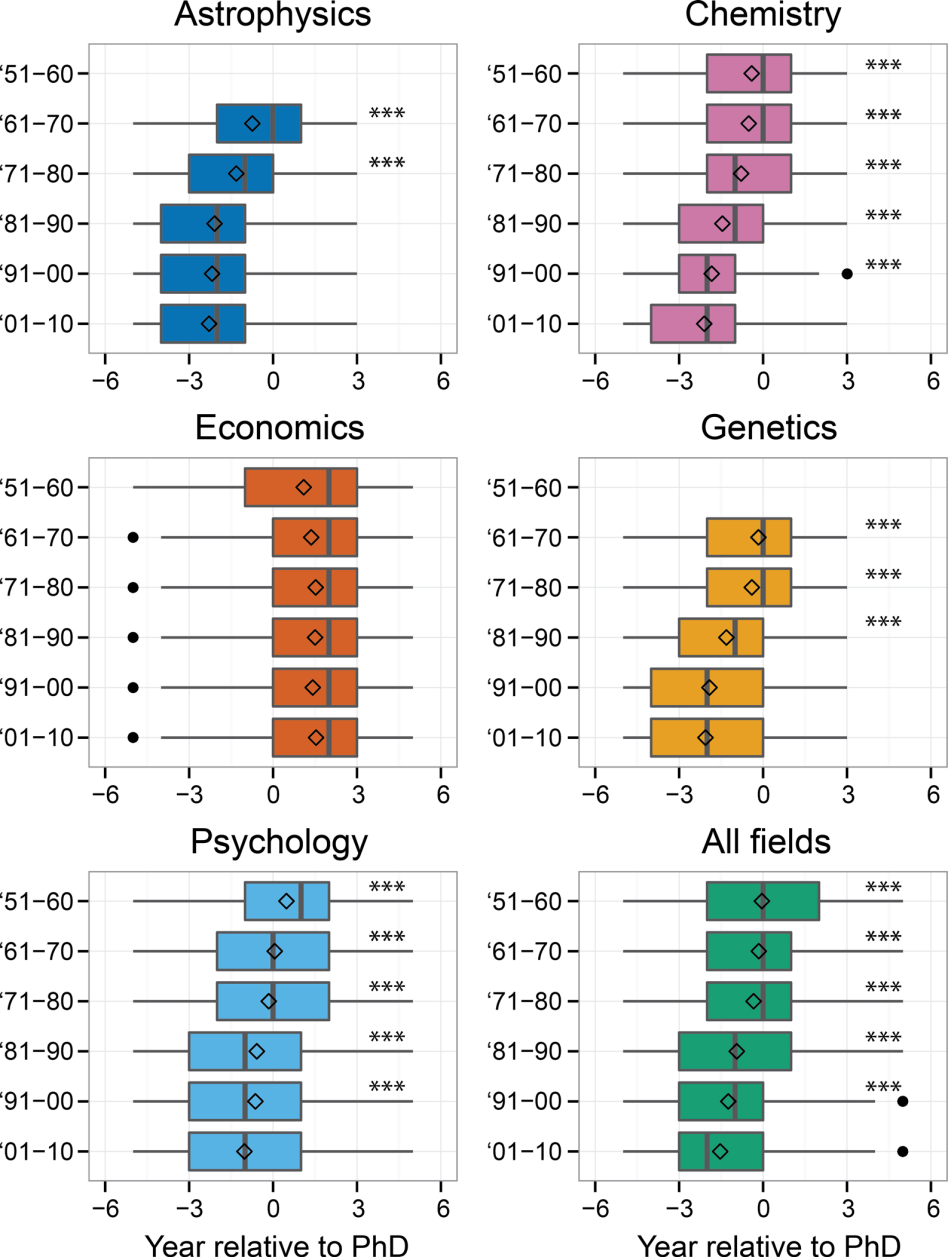
First publication relative to the year of PhD. For doctorate recipients with at least one publication, the relative year of their first publication was determined (with 0 being the year of PhD). Relative year of first publication is plotted by year of PhD (in ten-year periods; only plotted when number of doctorate recipients with at least one publication in a period > 50). The diamond represents the mean relative year of first publication. Stars denote the median of a group differs significantly from the ‘01-’10 group (p < 0.001 in Kruskal-Wallis test). The selection of doctorate recipients was restricted to having a first publication between five years before and three years after PhD (astrophysics, chemistry and genetics) or between five years before and five years after PhD (economics and psychology; seeData and methods section).

Year of first publication illuminates important trends in scholarly communication behaviors of doctoral students. For the U.S. doctorate recipients who published at least one article, we determined when they published it relative to when they received their PhD (Fig 2). As shown, in all fields except economics the year of first publication has shifted to before conferral of the PhD. The clearest shifts were observed in chemistry, genetics, and psychology: in the 1951–1960 period the median first publication was in the year of PhD or one year after, and in the 2001–2010 period, the median is one or two years *before* PhD. Only in economics did the relative year of first publication not change, remaining at two years after the doctorate.

### Post-PhD career spans of doctorate recipients

**Fig 3:**
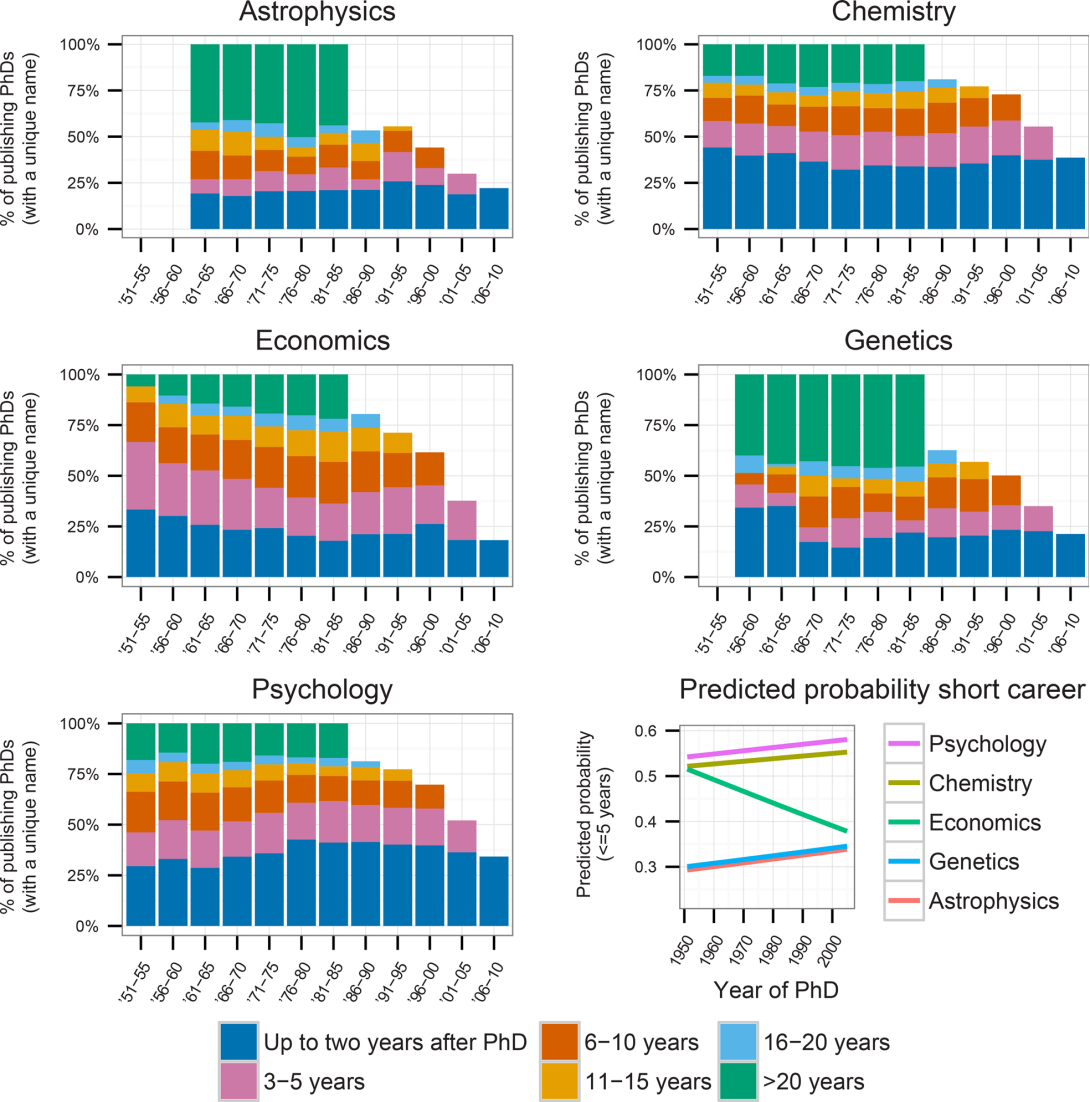
Career length by five-year period and field. Papers published after publications interruptions of over five years removed (only plotted when number of doctorate recipients with one or more published papers in a five-year period > 25, and when all doctorate recipients in a five-year period have had the opportunity to publish in a given period). Shortest careers are at the bottom of the stacked bars, the longest at the top.

Post-PhD career spans of U.S. doctorate recipients we examined by computing the share of recipients publishing at various career lengths: up to two, three to five, six to ten, eleven to fifteen, sixteen to twenty, and twenty to thirty years since the doctorate. We consider the publication career spans of the 1951–2010 doctorate recipients, with papers published after a long interruption in publication (five years or longer) removed (Fig 3). In such an investigation, no distinction can yet be made between post-1985 doctorate recipients with a short publication career and those whose publication careers have been interrupted but who will later resume publishing. For example, a scholar receiving a PhD in 1990 could have published his or her last paper in 1995, which means a career span of five years. However, he or she could publish a next paper in 2017, which would mean their career span would actually be 27 years. With our current data we naturally cannot measure 2017 publications. However, when papers published after an interruption are disregarded, the determination of post-1985 doctorate recipients’ publication career length can be performed. Results for a publication career category are only shown when all doctorate recipients in a five-year period have been followed for the entire span of the publication career category. An example: for 1986–1990 doctorate recipients, we do not plot the shares of doctorate recipients publishing for >20 years after PhD, because these recipients should then have been followed from the year of PhD until 2016 to 2010 (as the >20 years category is comprised of persons publishing for 20–30 years since PhD). Hence, the shares do not add up to 100%.

The main change in publication career length has been in the shares of doctorate recipients without any publications (S5 Fig), especially in astrophysics, psychology (where this share has increased), and in genetics (where it has decreased). As these shares are often quite high, it is difficult to study trends in the publication careers of publishing doctorate recipients. Furthermore, it is difficult to conclude whether increasing or decreasing shares of certain career spans are due to a relative lengthening or shortening of career spans, or due to changing shares of publishing doctorate recipients. Therefore, in our further analysis of career spans, we disregard doctorate recipients who have not published at all.

These results show that the share of doctorate recipients with a long publication career (over twenty years) is quite large, especially in astrophysics and genetics: about 40%. This share is lower in chemistry (about 20%), economics (between 5 and 20%), and psychology (between 15 and 20%).

Looking at trends in the spans of publication careers, in astrophysics, chemistry, genetics and psychology, the share of doctorate recipients with long publication careers has remained quite stable, but increased in economics. The share of intermediate length careers (6–20 years) decreased in psychology in the late 1980s and the 1990s. With respect to the share of brief publication careers, in chemistry, recent decades have seen a slight upward trend after an initial decline. In astrophysics, these shares increased from the 1970s to the 2000s, and in psychology, they increased from the 1950s until the 2000s, Conversely, in economics, there was a downward trend. To further investigate the probability of doctorate recipients having a short publication career, we predicted the probability of having a publication career lasting up to five years since PhD for 1951–2005 U.S. doctorate recipients, using a logit regression. The dependent variable was the dichotomous variable “publication career < = 5 years”, and the independent variables were year of PhD, field and an interaction term between the two. The predicted probabilities show that in all fields except economics, the probability of having a short publication career increased (Fig 3, bottom right panel). This shows that, although the span of publication careers remained relatively stable during the investigated period, the share of doctorate recipients with a short publication career slightly increased (except in economics). However, here it must also be noted that the positive slopes are in part due to the low numbers of doctorate recipients in earlier decades of our study, which thus have a small effect in the regression. For example, the slope of genetics is positive, although the shares of doctorate recipients with a short publication career (Fig 3, genetics panel) were actually larger in the 1950s and early 1960s than later. It is due to the small numbers of doctorate recipients in this period and the larger numbers later that the slope is positive.

## Discussion and Conclusion

The large growth in the number of doctoral students compared to a smaller growth in tenure track positions have raised concerns in the scholarly community [2, 7, 8, 40]. In a recent article, Ioannidis et al. argued that the current academic career system is recruiting too many doctoral students without being able to offer them long-term careers, and is in fact exploiting them [9]. This conclusion is supported by the fact they found only 1% of the scientific workforce to be continually publishing. We investigated whether the changes in academic employment have replaced long investigative careers with more volatile ones, and whether publication practices during the doctoral training period have changed.

We find that the proportion of publishing doctorate recipients has changed through time, but that this trend differs by field: in astrophysics, economics and psychology the proportion went down, in chemistry it remained quite stable, and in genetics it went up. Furthermore, the share itself varies by field; it is highest in astrophysics and lowest in economics. This difference is probably due to the nature of the fields. In the natural and life sciences, research is usually conducted in larger teams than in the social sciences, and graduate students granted authorship as the result of their role in such teams [8, 11].

The span of publication careers also varies by field, a finding also very likely to be related to the nature of the various fields. In the basic research fields of astrophysics and genetics many doctorate recipients continue to publish scientific findings for a long time. In contrast, in the professional and more applied fields of chemistry and psychology, doctorate recipients are more likely to find non-academic employment, leading to shorter publication careers. In economics, a field we characterized as more academic than psychology in our Data and methods section, publication careers are only slightly longer than in psychology. This may be due to the fact that we did not include articles published more than five years apart; as productivity levels in economics are lower than in the natural sciences, it is more likely that academic papers are published more than five years apart [11].

At the same time, the time of doctoral recipients’ first publication has shifted. In astrophysics, chemistry, genetics and psychology the year of first publication shifted from after the PhD to several years before the PhD. Only in economics did the first publication year remain stable. Several factors may explain this trend, such as research groups shifting their research tasks and activities from scientists with a PhD to those without, indicating an increasing reliance on doctoral students for the production of knowledge [4, 41, 42]. Another reason is an increasing focus on publication as part of PhD students’ so-called “socialization” to the world of research [43]. Finally, the increasing rates of collaboration in science may have led to more doctoral students being granted authorship on research conducted together with more senior colleagues [44, 45].

Going back to the question whether the relative decline of tenured and tenure track positions led to shorter investigative careers, our results show this is not truly the case. Although the probability of shorter careers has increased to a slight extent, the span of the publication career has remained quite stable (in astrophysics, chemistry, genetics, and psychology) or even increased (in economics). Furthermore, in the basic research fields of astrophysics and genetics, long publication careers (of over twenty years) have been the most common career for doctorate recipients from the early 1950s to the early 1980s. These findings seem to be at odds with the findings of Ioannidis et al.’s study of the share of scientists publishing one or more papers every year [9] and Petersen et al.’s study of career longevity in high-profile journals [46]. These differences in findings are likely due to the fact that we did not restrict our sample to scientists publishing every year but included scientists publishing once in five years. Most importantly, our selection of scientists differs from that of Ioannidis and colleagues. They look at *all* authors with a publication in the bibliographic database Scopus, which also includes technicians and undergraduate students. In contrast, we look at people who have received a PhD. This degree qualifies them for an academic position that enables them to have a longer publication career. Petersen et al. found that most authors have a very short publication career in high-profile journals [46]. However, as these authors also indicate, the publication career span of a scientist in a particular top tier journal is not the same as the entire career span of a scientist, which explains our dissimilar findings.

When interpreting our findings, it must be borne in mind that our findings are based on an analysis of the publication careers of doctorate recipients with rare names. As already noted earlier in the paper, this means doctorate recipients with common names are not included in the analysis, which is likely to lead to an underrepresentation of doctorate recipients of East Asian origin (primarily Chinese and Korean).

In conclusion, not only are long publication careers common, the shares of more recent doctorate recipients publishing for a short period after the PhD are also stable. Therefore, while employment structures may have changed, the span of research activity by doctorate recipients has not. So in what types of positions do these academics work? Data on academic positions show a large increase in the number of postdoctoral positions [47, 48]. This rise is due to both an increase in the number of recent doctorate recipients taking a first postdoctoral position and an increase in the time spent in postdoctoral positions [49]. In addition, the number of non-tenure track staff positions has increased through time [5]. Our results show researchers have publication careers that are as long as they were before. However, they may be in “holding positions” or on “soft money” (i.e., postdoctoral and non-tenure track positions) for a much longer time. In addition, they may have continued publishing in non-academic employment, although through time, the importance of industrial laboratories in basic research has actually decreased rather than increased, making this unlikely [50].

This leaves one final question: why were doctorate recipients not deterred by the relative lack of tenured and tenure track positions and did they continue to publish for the same length of time? The replacement of tenured and tenure track positions with short (postdoctoral) contracts lead to a larger effect of chance on academic career spans [51]. And one would expect diminished career prospects to lead to decreased attractiveness of science and more individuals opting to work outside science. Possibly, this is because the rewards are high if one *does* succeed at landing a tenured position, and one can then supervise and advise many junior researchers—the so-called “pyramid structure of science” [4]. Another reason may be the job characteristics that science offers. U.S. doctoral students expect academia to offer more freedom than industry [52] and doctorate recipients in the Netherlands and Denmark are attracted to academia because of the intellectual challenge, independence, and creativity offered, even though academia offers less job security [53, 54]. As such, the attractive force of science, in combination with the large rewards at the top of the career ladder, may enable science to retain comparable numbers of practitioners despite diminished career prospects.

## Supporting Information

S1 Fig Trends in awarded U.S. doctorates in NSF data and the ProQuest database. (A) Annual number of doctorate recipients according to National Science Foundation data [18, 19] and annual number of dissertations indexed in ProQuest. (B) Number of doctoral dissertations stored in ProQuest divided by number of doctorate recipients according to NSF data.
(TIF)

S2 Fig Annual number of doctorate recipients indexed in ProQuest, by field. (TIF)

S3 Fig Number of journals indexed in Web of Science, by field and year. (TIF)

S4 Fig Selection of U.S. doctorate recipients with unique names. The used parameters were selection for names (combination of surname and one of more initials) unique in ProQuest (“Unique name ProQuest”), having two or three initials (“Two/three initials”), having a rare surname according to WoS (having a surname that does not occur in more than 100 surname-initial combinations in WoS; “Frequency name WoS”), not publishing outside of the own field and related disciplines (“Field publications”), and having the first publication up to three (astrophysics, chemistry and genetics) or five years (economics and psychology; “First publication”). (A) Number of remaining doctorate recipients in the selected sample after each selection step. (B) Mean number of published papers by doctorate recipients in the selected sample after each selection step.
(TIF)

S5 Fig Career length by five-year period and field including non-publishing doctorate recipients. Papers published after publications interruptions of over five years removed (only plotted when number of doctorate recipients with one or more published papers in a five-year period > 25, and when all doctorate recipients in a five-year period have had the opportunity to publish in a given period).
(TIF)

S1 Table Share of women among U.S. doctoral recipients with unique names. (PDF)

S2 Table Broader research field for each group of doctorate recipients. (PDF)

S3 Table Share of doctorate recipients with a unique name relative to total number of doctorate recipients (for whom a name is given in ProQuest), by year and discipline (PDF)
