# Supplementary figures and images for: Stability and Longevity in the Publication Careers of U.S. Doctorate Recipients
Source: PLoS One. 2016 Apr 29;11(4):e0154741. doi: 10.1371/journal.pone.0154741 (PMC4851373; doi:10.1371/journal.pone.0154741)

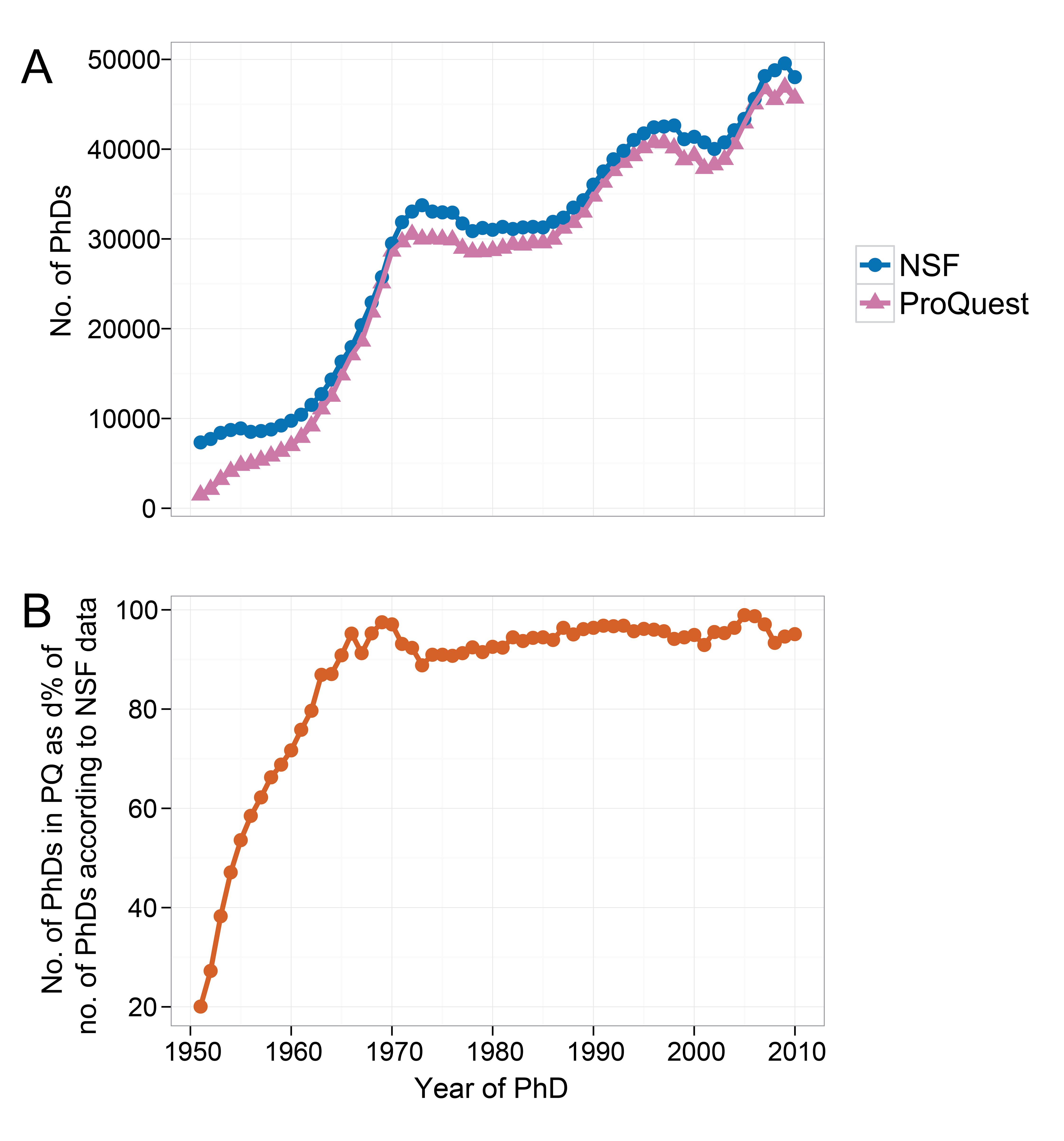

Supplement: S1 Fig — (A) Annual number of doctorate recipients according to National Science Foundation data [18, 19] and annual number of dissertations indexed in ProQuest. (B) Number of doctoral dissertations stored in ProQuest divided by number of doctorate recipients according to NSF data. (TIF) [file pone.0154741.s001.tif]

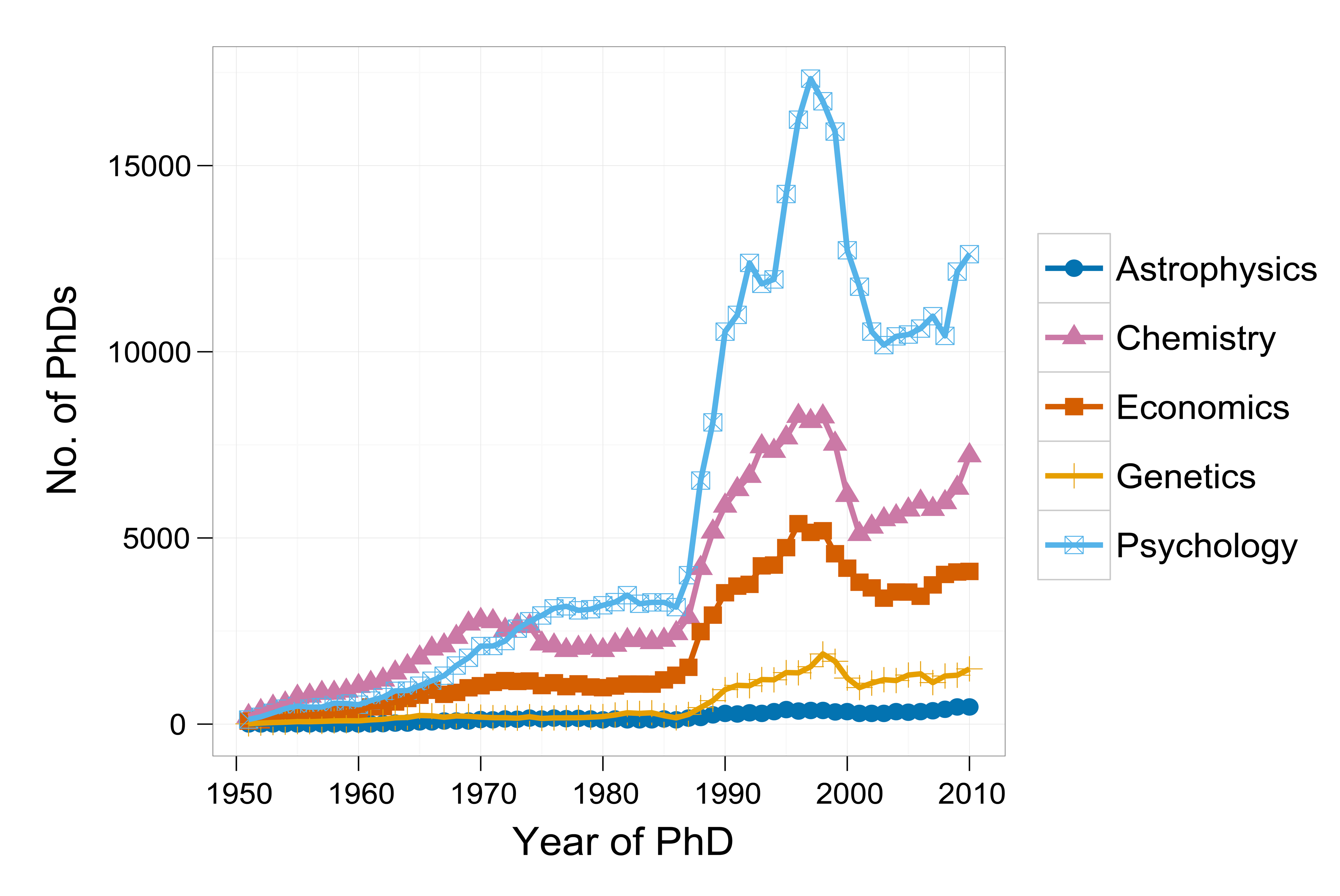

Supplement: S2 Fig — (TIF) [file pone.0154741.s002.tif]

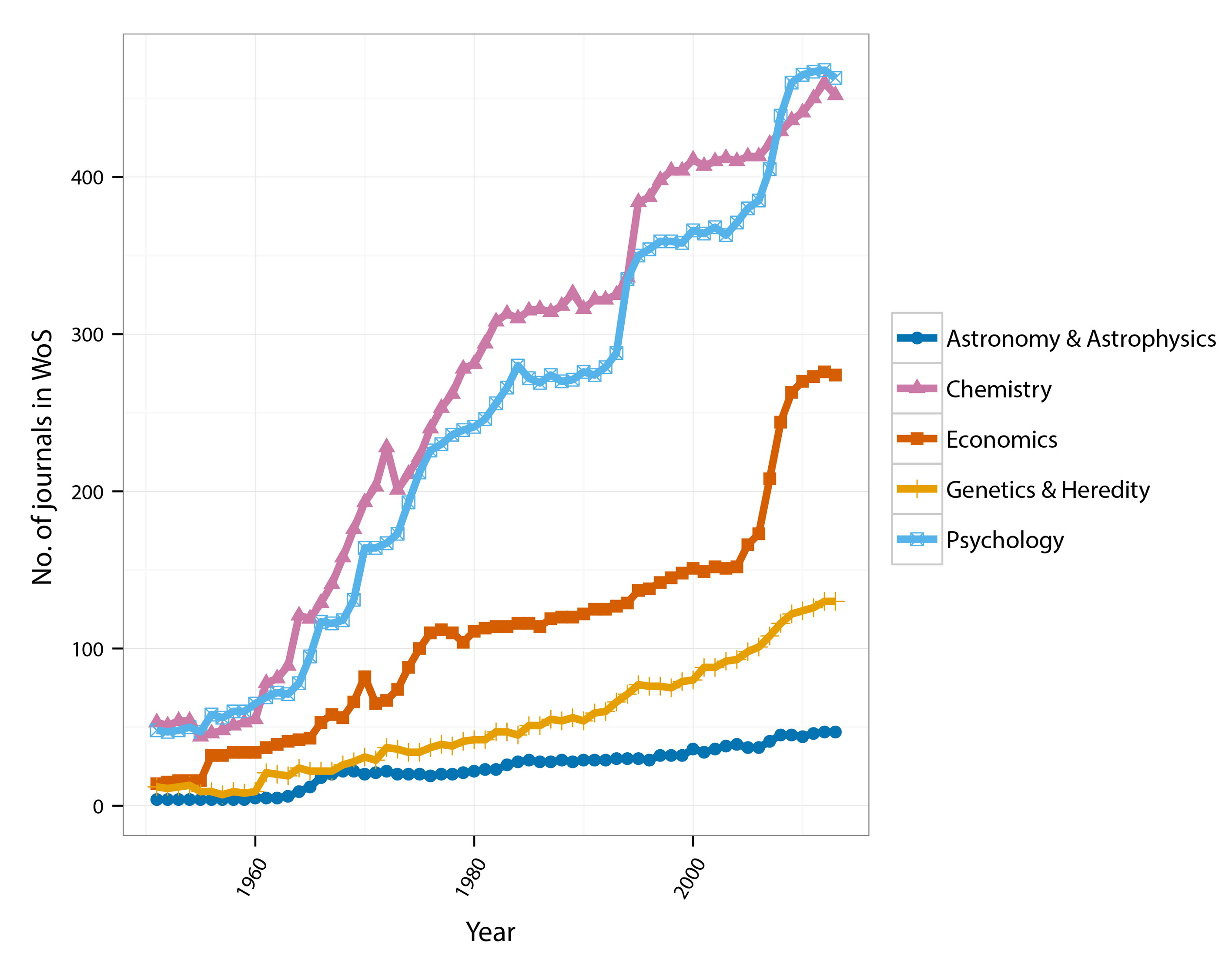

Supplement: S3 Fig — (TIF) [file pone.0154741.s003.tif]

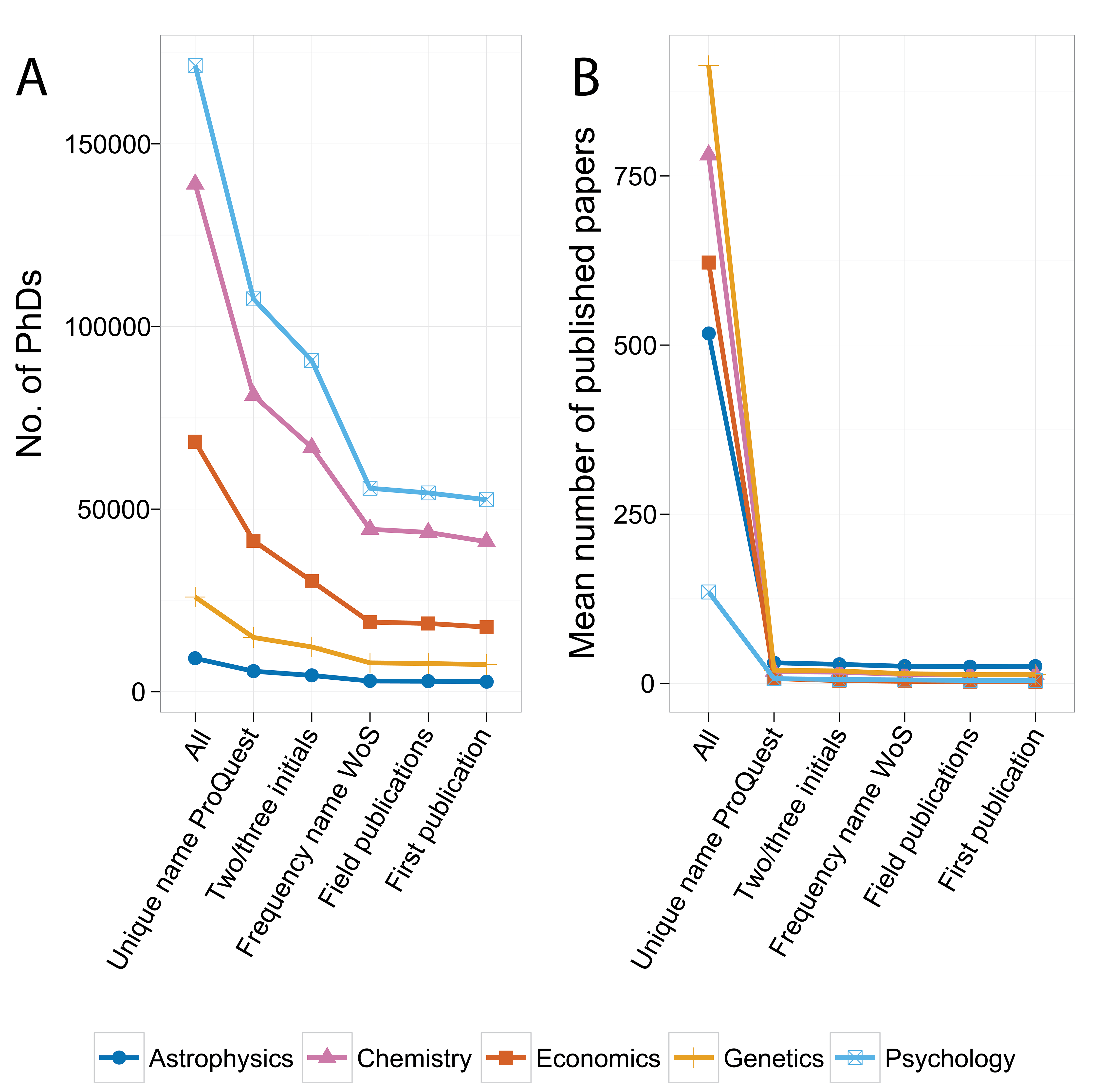

Supplement: S4 Fig — The used parameters were selection for names (combination of surname and one of more initials) unique in ProQuest (“Unique name ProQuest”), having two or three initials (“Two/three initials”), having a rare surname according to WoS (having a surname that does not occur in more than 100 surname-initial combinations in WoS; “Frequency name WoS”), not publishing outside of the own field and related disciplines (“Field publications”), and having the first publication up to three (astrophysics, chemistry and genetics) or five years (economics and psychology; “First publication”). (A) Number of remaining doctorate recipients in the selected sample after each selection step. (B) Mean number of published papers by doctorate recipients in the selected sample after each selection step. (TIF) [file pone.0154741.s004.tif]

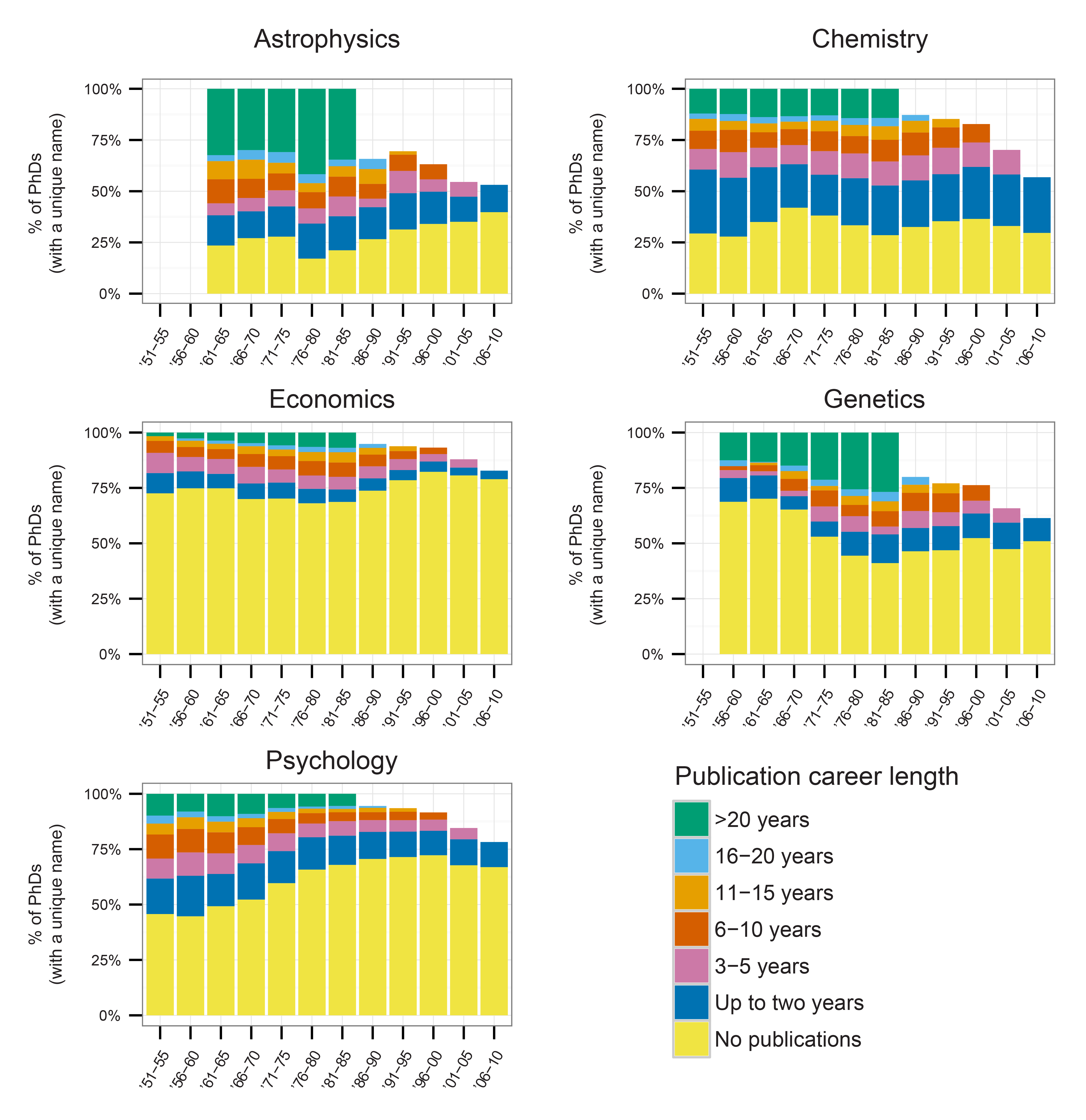

Supplement: S5 Fig — Papers published after publications interruptions of over five years removed (only plotted when number of doctorate recipients with one or more published papers in a five-year period > 25, and when all doctorate recipients in a five-year period have had the opportunity to publish in a given period). (TIF) [file pone.0154741.s005.tif]
