# Supplementary material for: Stability and Longevity in the Publication Careers of U.S. Doctorate Recipients
Source: PLoS One. 2016 Apr 29;11(4):e0154741. doi: 10.1371/journal.pone.0154741 (PMC4851373; doi:10.1371/journal.pone.0154741)
Supplement: S1 Table — (PDF) [file pone.0154741.s006.pdf]

S1 Table. Share of women among doctorate recipients with unique names

Astrophysics	Male	Female	Other
1951-1960	93%	0%	7%
1961-1970	90%	8%	2%
1971-1980	88%	8%	3%
1981-1990	83%	13%	5%
1991-2000	77%	16%	8%
2001-2010	67%	24%	9%
Total	78%	16%	7%

Chemistry	Male	Female	Other
1951-1960	89%	6%	5%
1961-1970	84%	9%	6%
1971-1980	80%	14%	6%
1981-1990	70%	22%	8%
1991-2000	62%	30%	8%
2001-2010	58%	34%	8%
Total	70%	22%	7%

Economics	Male	Female	Other
1951-1960	88%	5%	7%
1961-1970	86%	6%	9%
1971-1980	82%	9%	9%
1981-1990	71%	17%	11%
1991-2000	65%	25%	10%
2001-2010	59%	27%	14%
Total	71%	18%	10%

Genetics	Male	Female	Other
1951-1960	77%	13%	9%
1961-1970	71%	19%	10%
1971-1980	64%	29%	7%
1981-1990	59%	35%	7%
1991-2000	51%	41%	8%
2001-2010	46%	44%	10%
Total	53%	38%	9%

Psychology	Male	Female	Other
1951-1960	79%	16%	5%
1961-1970	75%	21%	4%
1971-1980	61%	34%	4%
1981-1990	45%	50%	5%
1991-2000	36%	61%	3%
2001-2010	30%	61%	9%
Total	44%	50%	5%
