# Supplementary material for: Stability and Longevity in the Publication Careers of U.S. Doctorate Recipients
Source: PLoS One. 2016 Apr 29;11(4):e0154741. doi: 10.1371/journal.pone.0154741 (PMC4851373; doi:10.1371/journal.pone.0154741)
Supplement: S2 Table — (PDF) [file pone.0154741.s007.pdf]

S2 Table. Broader research field for each group of doctorate recipients

Field of U.S. doctorate recipients	WoS citation index	Additional WoS subdisciplines
Astrophysics	SCIE	
Chemistry	SCIE	
Economics	SSCI and AHCI	Biology[a]<br>Clinical medicine<br>Earth and space<br>Engineering and technology<br>Mathemathics
Genetics	SCIE	Psychology<br>Health
Psychology		Psychology[b]<br>Arts<br>Biomedical research<br>Clinical medicine<br>Engineering and technology<br>Health<br>Humanities<br>Professional fields

Abbreviations: SCIE – Science Citation Index Expanded, SSCI – Social Science Citation Index, AHCI – Arts and Humanities Citation Index

[a] Defining this fairly broad range of disciplines and subdisciplines was necessary in order not to exclude economists in agricultural economics, health economics, environmental economics, and the more mathematical economics.

[b] This broad range was needed so not to exclude psychologists working in the more clinical fields, linguistics, visual or auditory processing, or social work
