# Supplementary material for: Stability and Longevity in the Publication Careers of U.S. Doctorate Recipients
Source: PLoS One. 2016 Apr 29;11(4):e0154741. doi: 10.1371/journal.pone.0154741 (PMC4851373; doi:10.1371/journal.pone.0154741)
Supplement: S3 Table — (PDF) [file pone.0154741.s008.pdf]

S3 Table. Share of doctorate recipients with a unique name relative to total number of doctorate recipients (for whom a name is given in ProQuest), by year and discipline

Year of PhD	Astrophysics	Chemistry	Economics	Genetics	Psychology
		%			
1951-1955	25	27	26	28	26
1956-1960	25	30	28	29	28
1961-1965	25	30	29	32	28
1966-1970	25	30	30	28	31
1971-1975	35	31	30	30	31
1976-1980	32	33	30	33	33
1981-1985	35	34	30	35	32
1986-1990	31	32	27	33	32
1991-1995	29	27	24	27	28
1996-2000	32	28	25	28	31
2001-2005	29	29	22	27	30
2006-2010	27	25	19	27	28
Total	30	29	26	29	30
